# Microstructure and biomechanical characteristics of bone substitutes for trauma and orthopaedic surgery

**DOI:** 10.1186/1471-2474-12-34

**Published:** 2011-02-02

**Authors:** Esther MM Van Lieshout, Gerdine H Van Kralingen, Youssef El-Massoudi, Harrie Weinans, Peter Patka

**Affiliations:** 1 Department of Surgery-Traumatology, Erasmus MC, University Medical Centre Rotterdam, P.O. Box 2040, 3000 CA Rotterdam, the Netherlands; 2 Orthopedic Research Laboratory, Department of Orthopaedics, Erasmus MC, University Medical Centre Rotterdam, P.O. Box 1738, 3000 DR Rotterdam, the Netherlands

## Abstract

**Background:**

Many (artificial) bone substitute materials are currently available for use in orthopaedic trauma surgery. Objective data on their biological and biomechanical characteristics, which determine their clinical application, is mostly lacking. The aim of this study was to investigate structural and *in vitro *mechanical properties of nine bone substitute cements registered for use in orthopaedic trauma surgery in the Netherlands.

**Methods:**

Seven calcium phosphate cements (BoneSource^®^, Calcibon^®^, ChronOS^®^, Eurobone^®^, HydroSet™, Norian SRS^®^, and Ostim^®^), one calcium sulphate cement (MIIG^® ^X3), and one bioactive glass cement (Cortoss^®^) were tested. Structural characteristics were measured by micro-CT scanning. Compression strength and stiffness were determined following unconfined compression tests.

**Results:**

Each bone substitute had unique characteristics. Mean total porosity ranged from 53% (Ostim^®^) to 0.5% (Norian SRS^®^). Mean pore size exceeded 100 μm only in Eurobone^® ^and Cortoss^® ^(162.2 ± 107.1 μm and 148.4 ± 70.6 μm, respectively). However, 230 μm pores were found in Calcibon^®^, Norian SRS^®^, HydroSet™, and MIIG^® ^X3. Connectivity density ranged from 27/cm^3 ^for HydroSet™ to 0.03/cm^3 ^for Calcibon^®^. The ultimate compression strength was highest in Cortoss^® ^(47.32 MPa) and lowest in Ostim^® ^(0.24 MPa). Young's Modulus was highest in Calcibon^® ^(790 MPa) and lowest in Ostim^® ^(6 MPa).

**Conclusions:**

The bone substitutes tested display a wide range in structural properties and compression strength, indicating that they will be suitable for different clinical indications. The data outlined here will help surgeons to select the most suitable products currently available for specific clinical indications.

## Background

Treatment of bone defects is a continuous challenge in skeletal trauma and orthopaedic trauma surgery. Bone graft represents the second most common transplanted tissue, with blood being number one [1]. Worldwide, more than 2.2 million bone grafting procedures are performed annually for the repair of bone defects in orthopaedic traumatology, neurosurgery, and dentistry [2-4]. Approximately 10% of all skeletal reconstructive surgical interventions require bone grafting [4]. Large defects resulting from, among others, trauma, infection, or tumor resection often do not heal spontaneously, and require surgical intervention. In addition, the treatment of posttraumatic skeletal complications such as delayed unions, nonunions, or malunions frequently require bone grafting. Variations in size or location of the defect, but also patient related factors such as age and disease status determine the therapeutic approach. Herein, bone grafts provide support, fill voids, and enhance the biological repair of the defect.

Autogenous bone, either cortical or cancellous, harvested from the patient's iliac crest is considered the gold standard graft. Autogenous bone is an excellent grafting material, since it provides three of the four critical elements for bone repair; an osteoconductive matrix that provides a scaffold for bone ingrowth, growth factors that stimulate osteoinduction, and living bone cells that offer osteogenesis [5]. However, as the cells do not necessarily survive transplantation, the clinical benefit is not guaranteed per se [6]. Several limitations have been noted, including a limited amount or inappropriate shape of the graft [1]. Also, the harvesting of autogenous bone tissue lengthens the surgical procedure, and is associated with an 8-39% risk of complications that include infection, blood loss, haematoma, nerve and urethral injury, fracture, pelvic instability, cosmetic disadvantages, postoperative pain, and morbidity and chronic pain at the donor site [1,7-14]. Finally, the use of autografts is not recommended in elderly or pediatric patients or in patients with a malignancy or infectious disease.

Alternative bone grafts like iso-, allo-, and xeno-transplants have been applied, but due to (major) disadvantages their use is discouraged (for review, see [1,8]).

The first use of plaster of paris (gypsum) as an artifical bone substitute was reported on in 1892 [15]. Technological evolution and a better understanding of bone-healing biology have led to the development of alternative (synthetic) bone substitutes. In the eighties, calcium phosphate salts such as tricalciumphosphate (TCP) and hydroxyapatite (HA) were introduced for clinical use [16]. Although they do not exist naturally, TCP and HA have been shown to induce a biologic response similar to that of bone [1]. Other groups of compounds available are calcium sulphate (gypsum), type I collagen and non-biologic substrates like degradable polymers and bioactive glass [1,17,18]. Over 20 bone substitute products are registered at present for use in orthopaedic trauma surgery in the Netherlands [19]. They differ in composition, characteristics, appearances, and delivery forms (*e.g*., pastes, solid matrices, or granules).

Availability of an increasing number of products may seem attractive; however, without sufficient knowledge on their properties and behavior *in vivo *it will become more and more complicated to select the product that mimics the bone to be replaced the best. Determining which product to use is based upon many factors including the size and location of the defect as well as the handling properties and ability to deliver the material to the surgical site. The structure and biomechanical characteristics of the products are critical to their success. For the majority of products, these data are mostly lacking. The aim of this study was to investigate the *in vitro *porosity, structure characteristics, and compression strength and stiffness of bone substitutes that were registered for use in orthopaedic trauma surgery in the Netherlands and were available as (injectable) paste. Standardized tests were performed.

## Methods

### Sample preparation

**Figure 1:**
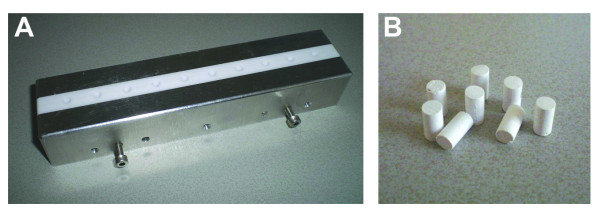
**Production of test samples **Test samples with a height of 8 mm and a diameter of 4 mm were made using a custom-made Teflon mold (panel A). Panel B shows examples of Calcibon^® ^test samples.

Nine bone substitutes that were available as (injectable) paste were selected for biomechanical testing; seven calcium phosphate cements, one calcium sulphate and one bioactive glass (Table 1). The products were stored at room temperature until use. Ten to 12 cylindrical test samples were prepared per product using a custom-made Teflon mould (Dept. Experimental Medical Instrumentation, Erasmus MC, Rotterdam, the Netherlands; Figure 1). Samples had a length of 8 mm and a diameter of 4 mm. This 2:1 ratio was the optimal ratio according Hing et al [20]. Samples were allowed to harden for 20 minutes at room temperature, after which micro-CT scanning was performed. Sample density was calculated from the length, diameter and weight. Subsequently, samples were kept at 37°C for 3 days in sterile water to allow for maximal hardening, upon which a compression test was performed.

**Table 1:** Bone substitutes tested for their biomechanical characteristics

Main ingredient	Product name	Producer
Calcium phosphate	BoneSource^®^	Stryker Nederland B.V.
	Calcibon^®^	Biomet Europe
	ChronOS^® ^Inject	Synthes, Inc
	Eurobone^®^	Surgical concepts
	HydroSet™	Stryker Nederland B.V.
	Norian SRS^®^	Synthes, Inc
	Ostim^®^	Hereaus
Calcium sulphate	MIIG^® ^X3	Wright Medical, Inc
Bioactive glass	Cortoss^®^	Orthovita, Inc

### Micro-CT scanning

Architecture was determined using a micro-CT (Skyscan 1076, Kontich, Belgium). The micro-CT was tuned at 70 kV and 140 μA, with a resolution of 9 μm. This setup was verified by scanning a Vitoss^® ^test sample with a known porosity between 88 and 92% [21], which was indeed within this range (data not shown). CT shadow projection images were converted into a three dimensional reconstruction of cross-sectional images in bitmap files using the volumetric reconstruction software (Nrecon software, Skyscan, Belgium). Total, closed and open porosity, connectivity density, structure model index (SMI) and pore size were calculated from these 3D reconstruction using the CTAn software (SkyScan, Kontich, Belgium). Total porosity was defined as the volume of all open plus closed pores as a percent of the total Volume Of Interest (VOI) volume. Closed porosity represents the volume of the closed pores as a percent of the total of solid plus closed pore volume within the VOI. Open porosity is defined as the volume of open pores as a percent of the total VOI volume. Connectivity density is the number of redundant connections between trabecular structures per unit volume. The SMI indicates the relative prevalence of rods and plates in a 3D structure. Pore size was defined as the average thickness of the pores, similar to the definition of trabecular spacing and thickness [22].

### Biomechanical testing

The compression strength was determined using unconfined compression tests. Upon five consecutive non-destructive preconditioning cycles, samples were compressed at a velocity of 0.5 mm/min to fracture using a standard compression-testing device (Lloyd Instruments, Fareham, UK). The resulting Extension-force curves were converted to Strain-stress curves using formulas I and II:

(I) Strain (mm/mm) = Extension/Lo

(II) Stress (MPa) = Force/Ao

Herein, Lo is the original length of the sample and Ao is area of the sample. The ultimate strength (MPa) was determined as the maximum force applied per square mm recorded during the experiment. Stiffness (Young's modulus; MPa) was determined as the slope of the linear fit detected during the test.

### Data analyses

Statistical analyses were performed using the Statistical Package for the Social Sciences (SPSS) version 16.0 (SPSS, Chicago, IL, USA). First, a One-Way Analysis of Variance (ANOVA) was performed to test the hypothesis that the mean value for a given parameter was equal for all products. Subsequently, post hoc pairwise multiple comparisons were performed using the Student's T-test, with Bonferroni correction for multiple testing. P-values < 0.05 were considered statistically significant.

## Results

### Sample characteristics

The average length and diameter were measured in order to check whether the test samples size was as intended. Results are shown in Table 2. The length ranged from 7.694 ± 0.104 mm (mean ± SD) for Ostim^® ^to 8.365 ± 0.085 mm for Eurobone^®^. The diameter ranged from 3.650 ± 0.103 mm for Ostim^® ^to 3.992 ± 0.047 for Calcibon^®^. Both the length and diameter of Ostim^® ^were statistically significantly less than the other products, implying that the Ostim^® ^samples had slightly shrunken (p < 0.001, Mann-Whitney U-test).

**Table 2:** Average length, diameter and weight of the test samples

	N	Length (mm)	Diameter (mm)	Weight (mg)
BoneSource^®^	10	8.225 ± 0.052	3.980 ± 0.035	181.8 ± 6.1
Calcibon^®^	12	8.271 ± 0.045	3.992 ± 0.047	179.5 ± 6.1
ChronOS^®^	10	8.265 ± 0.147	3.970 ± 0.059	174.5 ± 9.3
Eurobone^®^	10	8.365 ± 0.085	3.985 ± 0.053	186.5 ± 5.7
HydroSet™	10	8.325 ± 0.079	3.970 ± 0.042	179.8 ± 13.0
Norian SRS^®^	10	8.180 ± 0.079	3.915 ± 0.034	171.9 ± 2.6
Ostim^®^	9*	7.694 ± 0.104	3.650 ± 0.103	103.3 ± 7.2
MIIG^® ^X3	10	8.345 ± 0.064	3.985 ± 0.053	199.0 ± 5.4
Cortoss^®^	10	7.979 ± 0.103	3.854 ± 0.062	166.4 ± 4.6

Samples of bone substitute products were prepared using a custom-made Teflon mold as indicated in the Materials and Methods. The length and diameter were measured in order to confirm the intended size (8 mm length and 4 mm diameter). Data are presented as mean ± SD.

*, one test sample was discarded due to the presence of air bubbles.

The average weight of the test samples varied twofold. The lowest recorded mean weight was 103.3 ± 7.2 mg for Ostim^®^, whereas MIIG^® ^X3 had a weight of 199.0 ± 5.4 mg (Table 2).

**Figure 2:**
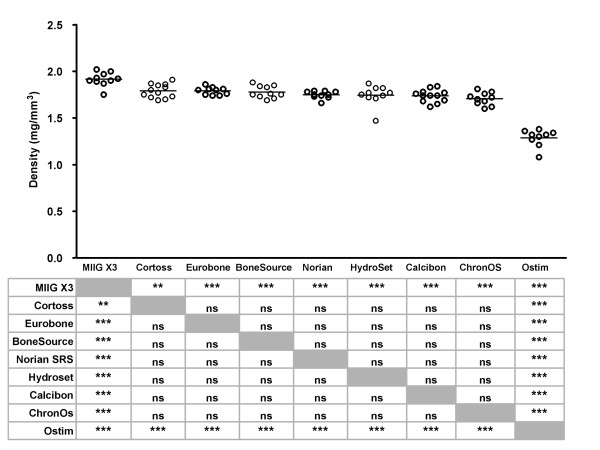
**Densities of bone substitutes **Densities of individual test samples were calculated from their length, diameter and weight. Each dot represents an individual test sample, and lines indicate the average value. The table below the figure provides an overview of the statistical analysis of pairwise comparisons (Student's T-test with Bonferroni correction). *, p < 0.05; **, p < 0.01; ***, p < 0.005; ns, not statistically significantly different. Grey boxes represent the self-self combinations, which could not be tested.

The density of all test samples was calculated from the length, diameter and weight (Figure 2). The CaSO_4 _MIIG^® ^X3 had the highest density (1.92 ± 0.08 mg/mm^3^), followed by the bioactive glass Cortoss^® ^and the CaPO_4 _Eurobone^®^, which both had an average density of 1.79 mg/mm^3^. The density of the other CaPO_4 _products ranged from 1.78 ± 0.07 mg/mm^3 ^(BoneSource^®^) to1.29 ± 0.09 mg/mm^3 ^(Ostim^®^). The density of MIIG^® ^X3 was significantly higher than all other products, whereas the density of Ostim^® ^was significantly lower.

### Porosity and pore size

**Figure 3:**
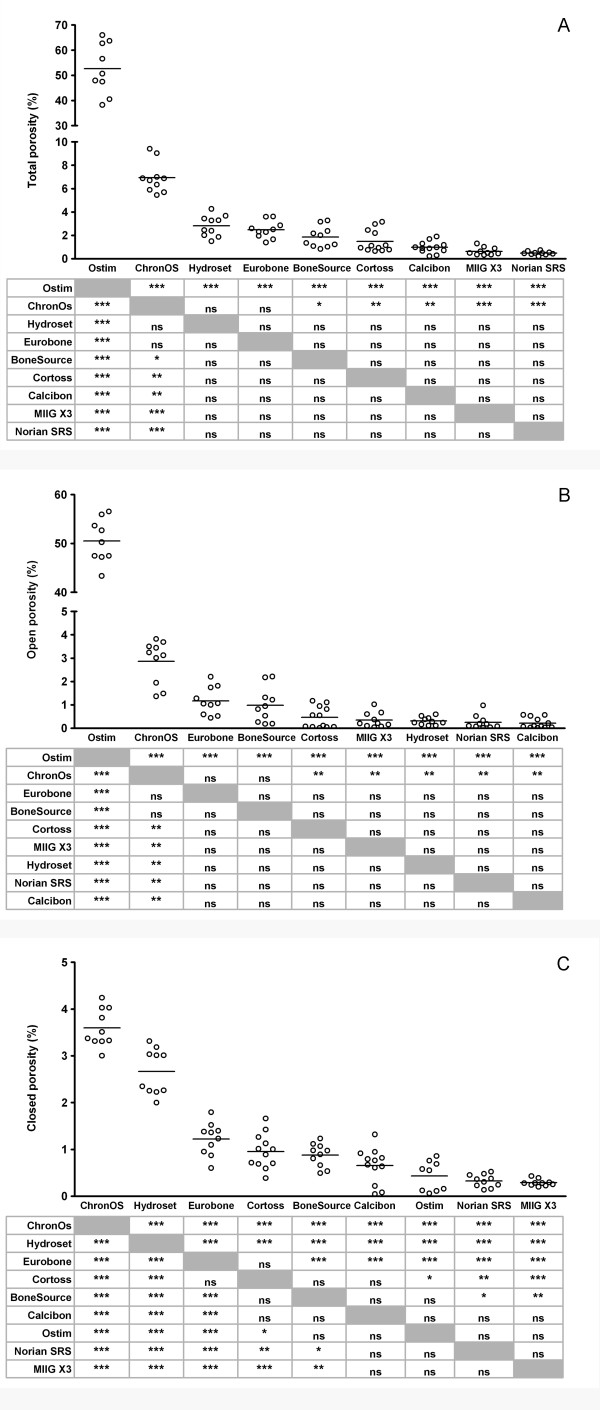
**Porosity of bone substitutes **Porosity of individual test samples was determined upon Micro-CT-scanning as described in the Materials and Methods. The total porosity (A), open porosity (B) and closed porosity (C) were determined. Each dot represents an individual test sample, and lines indicate the average value. The table below the figure shows the outcome of the pairwise comparisons (Student's T-test with Bonferroni correction). *, p < 0.05; **, p < 0.01; ***, p < 0.005; ns, not statistically significantly different. Grey boxes represent the self-self combinations, which could not be tested.

In order to gain insight into the porous structure of the bone substitute materials, the porosity and pore sizes were calculated from micro-CT images. Ostim^® ^was the only product that had a clear porous structure. The total porosity (52.66 ± 10.14%) was significantly higher than the porosity of all other products (Figure 3A). The porosity of the other products diminished from 6.93 ± 1.32% (ChronOS^®^) to 0.48 ± 0.15% for Norian SRS^®^. As total porosity is dictated by open as well as closed pores, the open porosity and closed porosity were also determined. Open porosity was evident for Ostim^® ^(50.52 ± 4.49%; Figure 3B), and diminished from 2.86 ± 0.92% (ChronOS^®^) to 0.22 ± 0.75% for Calcibon^®^. Closed porosity exceeded was highest for ChronOS^® ^(3.59 ± 0.41%) and HydroSet™ (2.66 ± 0.49%), and lowest for Ostim^® ^(0.43 ± 0.32%), Norian SRS^® ^(0.33 ± 0.13%), and MIIG^® ^X3 (0.29 ± 0.07%; Figure 3C).

**Figure 4:**
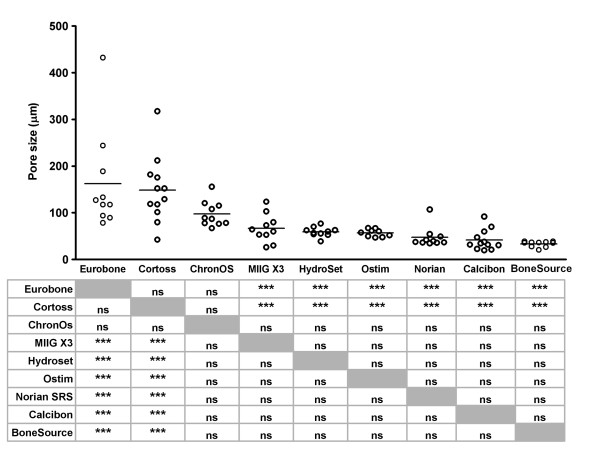
**Average pore sizes of bone substitutes **Average pore sizes of individual test samples were determined upon Micro-CT-scanning as described in the Materials and Methods. Each dot represents an individual test sample, and lines indicate the average value. The table below the figure shows the outcome of the pairwise comparisons (Student's T-test with Bonferroni correction). *, p < 0.05; **, p < 0.01; ***, p < 0.005; ns, not statistically significantly different. Grey boxes represent the self-self combinations, which could not be tested.

The porous structure of the bone substitute materials is determined by their porosity and pore size. Only two products had a mean pore size that exceeded 100 μm, *i.e*., 162.2 ± 107.1 μm for Eurobone^® ^and 148.4 ± 70.6 μm for Cortoss^® ^(Figure 4). Pore sizes of Norian SRS^® ^(47.2 ± 21.9 μm), Calcibon^® ^(41.6 ± 22.0 μm) and BoneSource^® ^(33.4 ± 6.2 μm) were below 50 μm.

**Figure 5:**
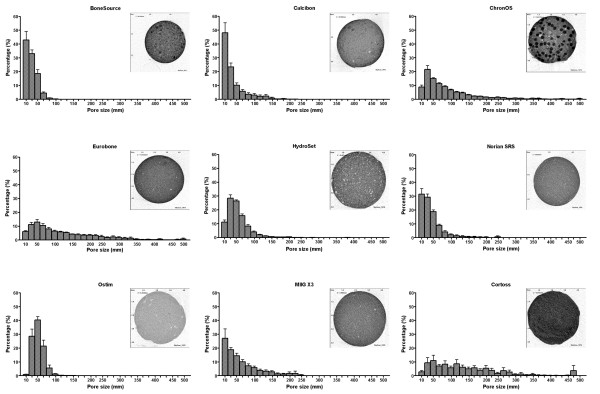
**Distribution of pore sizes of bone substitutes **The frequency of pore sizes varying from 10 to 500 μm are shown. Bars indicate the mean ± SD of the individual test samples (N = 9 to 12 per product). For each product, a typical example is given in the upper right corner of the panel.

For each product the range in pore sizes is shown in Figures 5. Of all products, BoneSource^® ^had the smallest pores. Over 95% of pores were smaller than 60 μm, of which approximately half were < 26.7 μm. No pores > 100 μm were found. This was also seen in Ostim^®^, of which 95% of pores were smaller than 85 μm. Calcibon^®^, Norian SRS^® ^and HydroSet™ incidentally showed pores up to 230 μm, however 95% were smaller than 125 μm. Of the CaPO4 products, ChronOS^® ^and Eurobone^® ^were the only two that contained pores up to 500 μm, with 95% of pores being smaller than 250 μm and 330 μm, respectively. The distribution of pore sizes of the CaSO_4 _MIIG^® ^X3 appeared similar as that of Norian SRS^® ^and, to a lesser extent, Calcibon^®^. However, with a maximum pore size of 250 μm and 90% of pores being < 190 μm, pores of MIIG^® ^X3 were relatively larger. The pore size frequency of Cortoss^® ^deviated from that of the other products tested, as a large range of pore sizes (25 to 300 μm) were approximately equally present. In this bioactive glass 95% of pores had sizes up to 390 μm, although pores of 500 μm were also found. Combining the data of total porosity and average pore size implied that bone substitute materials provide a wide range of products. Some had a high porosity with small pores (*e.g*., Ostim^®^), and at the other side of the spectrum products had a low porosity with large pores (*e.g*., Eurobone^®^).

### Connectivity density and structure model index

**Figure 6:**
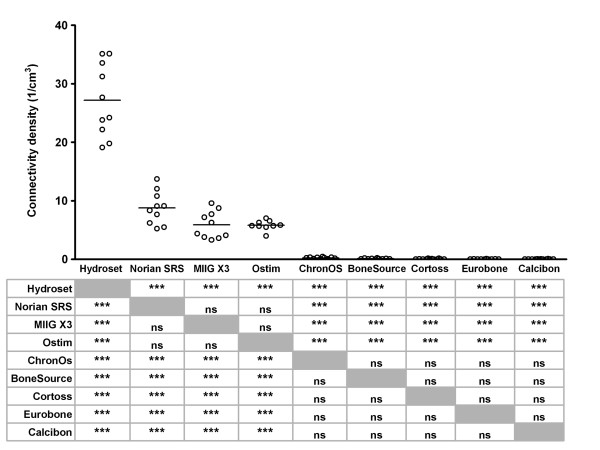
**Connectivity density of bone substitutes **The connectivity density of individual test samples was determined upon Micro-CT-scanning as described in the Materials and Methods. Each dot represents an individual test sample, and lines indicate the average value. The table below the figure shows the outcome of the pairwise comparisons (Student's T-test with Bonferroni correction). *, p < 0.05; **, p < 0.01; ***, p < 0.005; ns, not statistically significantly different. Grey boxes represent the self-self combinations, which could not be tested.

In order to further characterize the architecture of the bone substitutes, their connectivity density and structure model index were determined. The connectivity density was >25/cm^3 ^for HydroSet™ (27.17 ± 6.22/cm^3^), and between 5 and 10 for Norian SRS^®^, MIIG^® ^X3, and Ostim^® ^(8.77 ± 2.81, 5.87 ± 2.32, and 5.80 ± 0.84/cm^3^, respectively; Figure 6).

**Figure 7:**
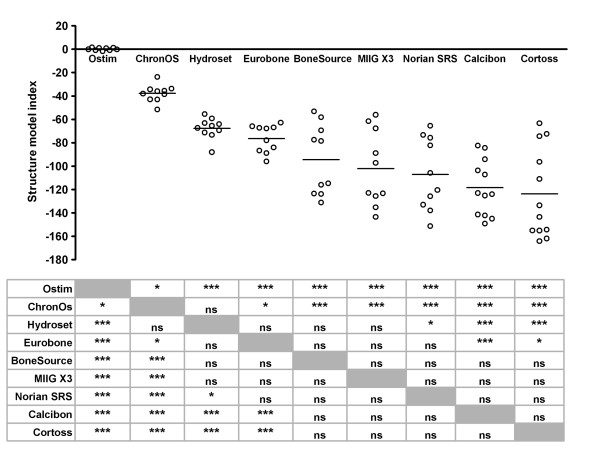
**Structure model index of bone substitutes **The structure model index of individual test samples was determined upon Micro-CT-scanning as described in the Materials and Methods. Each dot represents an individual test sample, and lines indicate the average value. The table below the figure shows the outcome of the pairwise comparisons (Student's T-test with Bonferroni correction). *, p < 0.05; **, p < 0.01; ***, p < 0.005; ns, not statistically significantly different. Grey boxes represent the self-self combinations, which could not be tested.

Ostim^® ^was the only product with a positive structure model index (SMI) (0.125 ± 1.165; Figure 7). For the other products, the SMI declined from -37.715 ± 7.280 for ChronOS^® ^and -67.752 ± 8.913 for HydroSet™ to -123.717 ± 38.232 for Cortoss^®^.

### Compression strength and Young's modulus

**Figure 8:**
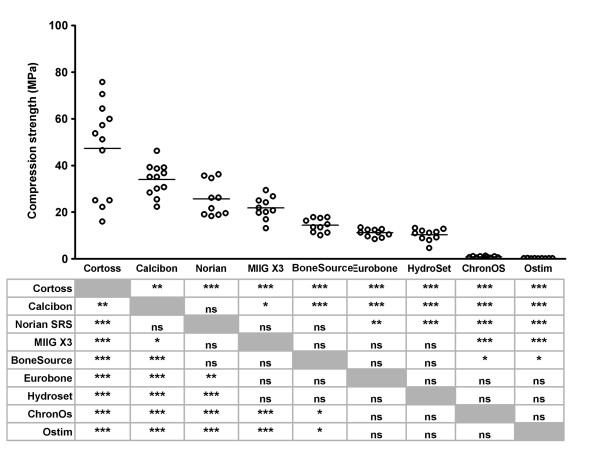
**Compression strength of bone substitutes **The compression strength was determined using unconfined compression tests as described in the Materials and Methods. Each dot represents an individual test sample, and lines indicate the average value. The table below the figure shows the outcome of the pairwise comparisons (Student's T-test with Bonferroni correction). *, p < 0.05; **, p < 0.01; ***, p < 0.005; ns, not statistically significantly different. Grey boxes represent the self-self combinations, which could not be tested.

The compression strength of all products was determined using unconfined compression tests. Cortoss^® ^had the highest ultimate compression strength (47.32 ± 20.34 MPa; see Figure 8). This was statistically significantly higher than the strength of all other products. Next in order of diminishing strength were Calcibon^® ^and Norian SRS^® ^(33.95 ± 6.75 and 25.64 ± 7.37 MPa, respectively), which was statistically significantly higher than most other products. ChronOS^® ^and Ostim^® ^had poor compression strengths (0.81 ± 0.32 and 0.24 ± 0.05 MPa, respectively).

**Figure 9:**
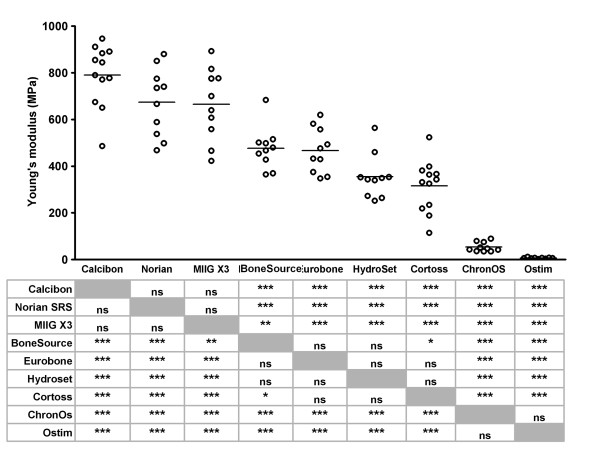
**Young's modulus of bone substitutes **The Young's modulus was determined using unconfined compression tests as described in the Materials and Methods. Each dot represents an individual test sample, and lines indicate the average value. The table below the figure shows the outcome of the pairwise comparisons (Student's T-test with Bonferroni correction). *, p < 0.05; **, p < 0.01; ***, p < 0.005; ns, not statistically significantly different. Grey boxes represent the self-self combinations, which could not be tested.

Calcibon^® ^had the highest Young's modulus (790 ± 132 MPa; Figure 9), followed by Norian SRS^® ^and MIIG^® ^X3 (674 ± 146 MPa and 665 ± 154 MPa, respectively). The Young's modulus of these three products was statistically significantly higher than that of the other products. ChronOS^® ^and Ostim^® ^had a very low Young's modulus (54 ± 20 MPa and 6 ± 3 MPa, respectively), which was statistically significantly lower than all other products.

## Discussion

Osteoconductive porous biomaterials provide a scaffold for the ingrowth of bone. With respect to pore size, microporosity (*i.e*., pores with a size < 5 μm) is considered important for the bioresorbability of the material [23], whereas macroporosity (*i.e*., pores > 100 μm) plays an important role in the osteoconductivity. A large macroporosity (*i.e*., 400-600 μm) facilitates infiltration by fibrovascular tissue and revascularization, thereby allowing for bone reconstruction. Investigations of bone ingrowth into porous materials with varying pore size have led to the consensus that the optimal pore radius for bone ingrowth is >50 μm and perhaps as large as 150 μm [24-27]. Of the bone substitute materials tested, Eurobone^®^, Cortoss^®^, and ChronOS^® ^could be considered as truly osteoconductive in terms of pore size, as they contain a considerable number of pores with sizes of up to 500 μm. For ChronOS^® ^the presence of pore sizes between 100 and 400 μm have been shown before [28,29]. Pore sizes between 100-250 μm are only marginally present in Calcibon^®^, HydroSet™, MIIG^® ^X3, and Norian SRS^®^. BoneSource^® ^and Ostim^® ^do not contain pores with a size of at least 100 μm, so based upon this *in vitro *measurement, these might not be considered as highly osteoconductive based upon pore size alone. This is in agreement with literature data available for BoneSource^® ^(2-50 μm) [30] and Calcibon^® ^(<1 μm) [31].

The invasion by host tissue is mostly facilitated by a larger porosity. Of the products tested, Ostim^® ^is the only product with a high mean total porosity of approximately 53%. Porosity of all other products is below 7%, with Norian SRS^® ^being the densest (0.48% mean total porosity). The total porosity as found for Cortoss^® ^(1.48 ± 0.94%) is in line with the 1% mentioned by the supplier. It is unclear why porosities of some other products were lower than previously published data, indicating a porosity of 60-75%) for ChronOS^® ^[28,29], 46% for BoneSource^® ^[32,33], and 30-40% for Calcibon^® ^[31,34]. This difference is unlikely to be due to an inadequate test design, since the porosity of 88-92% as found for the Vitoss^® ^test sample was exactly as previously shown [21]. As pores with a diameter below 9 μm could not be detected due to resolution restrictions of the CT scanner used in the present study, it cannot be ruled out that porosity and pore sizes are (slightly) under-estimated or overestimated, respectively. However, this unlikely explains differences between our data and literature data.

Adequate pore volume alone is not sufficient for achieving osteoconduction. Pore connectivity may determine the effectiveness of porosity [20,24,35-42]. In general, biomaterials with interconnected pores are considered to be superior to biomaterials containing closed pores, as interconnecting fenestrations provide the space for vascular tissue required for continued ingrowth of mineralized bone [27,35,36]. White & Shors indicated that such pore interconnections must be larger than 100 μm [37]. Of the products tested in this study, HydroSet™, Norian SRS^®^, MIIG^® ^X3, and Ostim^® ^had more than five interconnected pores per cm^3^. Connectivity density of the other products was 0.23/cm^3 ^or less. A negative correlation was found between pore size and connectivity density (Pearson correlation, r_p _= -0.21, p = 0.043), indicating that products with a lower pore size had a higher representation of interconnected pores.

The structure model index (SMI) indicates the relative prevalence of rods and plates in a 3D structure. SMI involves a measurement of surface convexity. Concave surfaces of enclosed cavities represent negative convexity to the SMI parameter. SMI values of ideal plates, cylinders and spheres are 0, 3, and 4, respectively. With a mean SMI value <0.2 Ostim^® ^appears to be mainly composed of plates. It is known that products with a total porosity below 50% often have a negative SMI. In this study, that was the case for eight out of nine products. Overall, SMI is positively correlated with total porosity, open porosity, and closed porosity (r_p _= 0.672, 0.645 and 0.358, respectively; p < 0.001), and negatively with compression strength and Young's Modulus (r_p _= -0.679 and -0.638, respectively, p < 0.001).

Although faster ingrowth is favoured by a more porous and interconnected structure, denser ceramics have better mechanical integrity [20,24,27]. For example, an increase of the total porous volume from 10% to 20% can result in a four-fold decrease in mechanical strength [27,43,44]. Of the bone substitute products tested, this phenomenon is most pronounced for Ostim^®^. Ostim^® ^has the highest total porosity (mean ~53%), but has poor compressive strength (mean 0.24 MPa) and Young's modulus (6 MPa). Calcibon^® ^and Norian SRS^®^, on the other hand, have low porosity (0.93% and 0.48%, respectively), but display a relatively high compressive strength (33.9 MPa and 25.6 MPa, respectively). Our data are in line with previous measurements, which revealed a compression strength of 6.3-34 MPa for BoneSource^® ^[45,46], 35-55 MPa for Calcibon^® ^[34,47], 14-24 MPa for HydroSet™ [48], and 23-55 MPa for Norian SRS^® ^[49-51]. For MIIG^® ^X3, an *in vivo *compression strength of 0.6 MPa has been shown at 13 weeks follow up in a canine fracture model [52]. This is lower than the 21.82 ± 21.93 MPa found in the current *in vitro *study, and is most likely due to a high degree of biodegradation and resorption of the MIIG^® ^X3 graft, as calcium sulphates are generally resorbed within 8-10 weeks. The 91-179 MPa as published for Cortoss^® ^[53] is higher than we found. This may be due to the larger size of the test samples (*i.e*., 8 × 7.5 × 100 mm) in the study by Boyd *et al*. [53]. As size and shape of the tested samples as well as the test setup itself may influence the outcome of the compression test, our data may allow for a more objective comparison of strengths between the products.

Overall, compression strength was negatively correlated with total porosity (r_p _= -0.424, p < 0.001), open porosity (r_p _= -0.399, p < 0.001), closed porosity (r_p _= -0.412, p < 0.001), and connectivity density (r_p _= -0.220, p = 0.034) (data not shown). Likewise, Young's modulus was negatively correlated with total, open and closed porosity (r_p _= -0.573, -0.539 and -0.491, respectively, p < 0.001). As opposed to porosity, pore size was unrelated to the compression strength (r_p _= 0.113, p = 0.281) or the Young's modulus (r_p _= -0.204, p = 0.050; data not shown).

The compounds tested in the current study represent the major classes of artificial bone grafts, *i.e*., calcium phosphates, calcium sulphate, and bioactive glass. Although selected based upon their availability in the Netherlands, their wide availability makes the data presented in this study generally relevant to most countries. Synthetic calcium phosphate cements can be moulded to irregularly shaped defects, or even injected via syringe before they harden *in situ*. The two main forms of calcium phosphates currently used are beta-tricalcium phosphate (β-TCP) and hydroxyapatite (HA), which can be used separately or combined in composite cements. Due to a general lack of macroporosity calcium phosphate cement degrades layer by layer from the outside to the inside. HA-cements tested include such as Ostim^® ^and HydroSet^® ^have a limited resorption rate. They are characterized by a high porosity, but a relatively low compressive strength. Clinical indications for Ostim^® ^include fractures of the tibia plateau [54,55], calcaneus [54], and distal radius [54,56,57]. There are currently no publications on clinical use of HydroSet™.

β-TCP has a compressive strength similar to that of cancellous bone [58], which may allow earlier weight bearing. However, it has a relatively high resorption rate. The β-TCPs ChronOs™ is mostly used in vertebral augmentation [59].

Combining HA and β-TCP improves the porosity of HA cement paste following implantation, because macropores are introduced into the HA composite after passive resorption of the β-TCP component. Subsequently, active resorption by monocytes/macrophages and osteoclasts can take place. Overall, calcium phosphate cement offers the highest mechanical compressive strength of any of the osteoconductive bone graft substitutes. Products such as BoneSource^®^, Calcibon^®^, ChronOs^® ^Inject, HydroSet™, and Norian SRS^® ^are densely packed at first, but will develop a porous network following resorption of the β-TCP component. Norian SRS^® ^and BoneSource^® ^have been studied the most; their clinical indications include fractures of the femur [60-63], tibia plateau [60,64,65], calcaneus [60,66,67], humerus [60,68], and distal radius [60,69-72]. Calcibon^® ^is mostly used in vertebral augmentation [73-75]. There are currently no publications on the clinical use of Eurobone^®^.

Of the available osteoconductive bone graft substitutes, calcium sulphate is the most rapidly resorbed. Because of its rapid resorption rate and low mechanical strength, calcium sulphate is recommended as a bone graft extender rather than as void filler. Clinical indications of MIIG^® ^X3 include fractures of the distal tibia and tibia plateau [76,77].

Bioactive glass possesses superior mechanical strength compared with calcium phosphate products, as a result of strong graft-bone bonding [1]. It is mainly used in craniofacial reconstructive surgery, dental, and orthopaedic trauma surgery. Cortoss^® ^is a low-viscosity glass-based cement that has been used successfully in fractures of the distal radius [78] and in vertebral augmentation [79,80].

The current study is restricted to biomechanical testing of bone substitute materials *in vitro*. As a next step, the biological behavior of these products *in vivo *should be determined in a standardized, comparative study. Pastes may harden less quickly in an aqueous dispersion *in vivo*, which may affect it ultimate strength. Combining data of our previous systematic review [19] with the data of the current *in vitro *study and a future *in vivo *study will allow for the development of a clinical guideline.

## Conclusions

The nine bone substitutes studied each have their individual characteristics, and provide orthopaedic trauma surgeons with a choice of products that varies largely in architecture and strength. Only for Eurobone^® ^and Cortoss^® ^the pore sizes exceed the 100 μm that is regarded necessary for proper osteoconduction. Biological and biomechanical characteristics of bone substitutes determine their applicability and success rate. Therefore, the *in vivo *behavior of these compounds (*e.g*., resorption rate and quality in bone ingrowth) should be taken into account as well. In general, bioactive glass will not resorb, and HA cements will remain in place for years. On the other hand, calcium sulphate cements may disappear before bone ingrowth has taken place. Calcium phosphate cements are generally densely packed and, consequently, provide more mechanical strength. The data outlined here will assist surgeons in selecting the most suitable product for specific clinical indications. Further studies on their *in vivo *behavior are needed for developing clinical guidelines for use of alternative bone substitute materials in orthopaedic trauma surgery.

## Competing interests

The authors declare that they have no competing interests.

## Authors' contributions

EMMVL, HW and PP designed the study. GHVK and YEM prepared all test samples and conducted the measurements. GHVK, YEM, and EMMVL extracted micro-CT data. EMMVL performed statistical analysis and designed all Figures and Tables. HW and PP critically revised the manuscript. All authors have read and approved the final manuscript.

## Pre-publication history

The pre-publication history for this paper can be accessed here:

http://www.biomedcentral.com/1471-2474/12/34/prepub
